# The 12 November 2025 Ugly Duckling Geomagnetic Storm: From the Sun to the Earth

**DOI:** 10.3390/s26051490

**Published:** 2026-02-27

**Authors:** Yury Yasyukevich, Ekaterina Danilchuk, Aleksandr Beletsky, Egor Borvenko, Aleksandr Chernyshov, Victor Fainshtein, Vera Ivanova, Denis Khabituev, Marina Kravtsova, Alexey Oinats, Sergey Olemskoy, Artem Padokhin, Konstantin Ratovsky, Valery Sdobnov, Artem Vesnin, Anna Yasyukevich, Sergey Yazev

**Affiliations:** 1 Institute of Solar-Terrestrial Physics of Siberian Branch of Russian Academy of Sciences, Irkutsk 664033, Russia; danilchuk.k@mail.ru (E.D.); beletsky@iszf.irk.ru (A.B.); borvenkoegor@gmail.com (E.B.); vfain@iszf.irk.ru (V.F.); moshkova@iszf.irk.ru (V.I.); khabituev@iszf.irk.ru (D.K.); rina@iszf.irk.ru (M.K.); oinats@iszf.irk.ru (A.O.); osv@iszf.irk.ru (S.O.); ratovsky@iszf.irk.ru (K.R.); sdobnov@iszf.irk.ru (V.S.); artem_vesnin@iszf.irk.ru (A.V.); annpol@iszf.irk.ru (A.Y.); syazev@gmail.com (S.Y.); 2 Faculty of Physics, Irkutsk State University, Irkutsk 664003, Russia; 3 Space Research Institute of Russian Academy of Sciences, Moscow 117997, Russia; achernyshov@cosmos.ru; 4 Faculty of Physics, Lomonosov Moscow State University, Moscow 119991, Russia; padokhin@physics.msu.ru; 5 Pushkov Institute of Terrestrial Magnetism, Ionosphere and Radio Wave Propagation of Russian Academy of Sciences, Moscow 108840, Russia

**Keywords:** geomagnetic storm, ionosphere, super equatorial plasma bubble, positioning errors, cosmic rays

## Abstract

**Highlights:**

**What are the main findings?**

- In November 2025, a series of consecutive coronal mass ejections associated with X-class solar flares from AR 14274 caused a severe geomagnetic storm (Kp = 9-, Dst = −217 nT and SYM-H = −254 nT). This resulted in the enhancement (up to 175 TECU) and poleward shift (8–10°) of equatorial anomaly crests, an equatorward-shifted auroral oval, and the appearance of SAR arcs and auroras at mid-latitudes.
- During the 12 November 2025 G4 geomagnetic storm, a super equatorial plasma bubble was recorded almost reaching the auroral oval boundary in the American sector.

**What are the implications of the main findings?**

- GPS kinematic Precise Point Positioning errors increased to 2–3 m at high latitudes and in regions affected by the equatorial bubble.
- During the main phase, a shift in the auroral oval resulted in radio aurora and signal absorption, which limited the potential of high-frequency radars at mid-latitudes.

**Abstract:**

The 12 November 2025 G4 geomagnetic storm—the third most intense of solar cycle 25—was triggered by a complex shock-ICME (interplanetary coronal mass ejection) structure as a result of three ICMEs and driven shocks that arrived on 11–12 November. The main enhancement in the interplanetary magnetic field occurred in the sheath region behind the shock driven by the second ICME. The Dst index reached −217 nT (the SYM-H index reached −254 nT) and the maximum Kp index was 9-. To comprehensively analyze the causes of the storm and its complex effects on near-Earth space, we used a multi-instrumental data set, involving data from satellite missions (ACE, SDO, PROBA2), GNSS networks, ionosondes, optical instruments, high-frequency radars (SuperDARN-like), and cosmic ray monitors. The auroral oval expanded equatorward (down to ~35° N in America). We recorded a super equatorial plasma bubble that almost reached the auroral oval boundary. The equatorial anomaly crests intensified, exceeding 175 TECU, and shifted poleward (8–10°). At mid-latitudes, the F2 layer critical frequency exhibited a strong negative disturbance (−50%) during the main phase, followed by an unusually prolonged and intense positive phase (+100%). GPS Precise Point Positioning errors increased to 2–3 m at high latitudes and in regions affected by the equatorial bubble. The event also featured a Forbush decrease and ground-level enhancement (GLE 77 according to the database hosted by the University of Oulu) associated with the X5.1 solar flare. The results underscore the complex chain of processes from solar storm to geomagnetic and ionospheric responses, highlighting the risks to satellite-based navigation and communication systems.

## 1. Introduction

Geomagnetic storms are one of the most significant factors of space weather. Complex processes of interaction between the atmosphere, ionosphere and magnetosphere occur during geomagnetic storms, causing various effects: the auroral oval expands equatorward, the electron concentration in the ionosphere changes [1,2], and ionospheric inhomogeneities of various scales appear [3,4]. These processes significantly impact the quality of technical systems [5], including radio equipment [6], global navigation satellite systems [7,8], and others [9,10].

Severe (class G4, Kp = 8–8.667, Dst < −200 nT) and extreme (class G5, Kp = 9, Dst < −350 nT) geomagnetic storms rarely occur [7]. Limited statistics and the significant differences between severe and extreme storms prevent us from making satisfactory predictions of such rare but dangerous events. Therefore, each new severe or extreme storm is of great interest. A severe geomagnetic storm occurred on 12 and 13 November 2025. The Kp index, which measures planetary disturbance in the geomagnetic field, reached 8.667, corresponding to a severe G4 storm, just short of the maximum mark.

**Figure 1:**
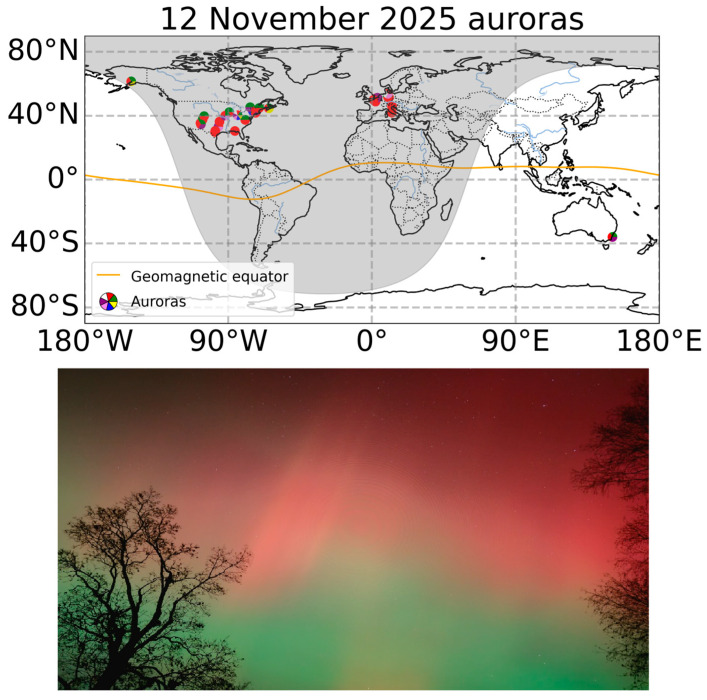
Map of auroras observed on 12 November 2025 (**top panel**) and photo of an aurora in the US (40° N, 84° W) on 12 November 2025 at 01:30 UTC (**bottom panel**). Photo credit: Lee Ann Kilgore, https://www.spaceweatherlive.com/en/archive/2025/11/12/observations (accessed on 2 February 2026). The solar terminator in the top panel corresponds to 02:00 UT on 12 November 2025.

Extreme geomagnetic storms often take their names after the dates of the associated solar flare, coronal mass ejection (CME), or geomagnetic disturbance onset/peak. The Bastille Day Solar Storm was named after the flare and CME that occurred on 14 July 2000 [11], the St. Patrick’s Day 2015 geomagnetic storm was named after the storm onset and Dst index minimum that occurred on 17 March 2015 [12], and the Halloween 2003 solar storm is associated with the prolonged solar and geomagnetic activity on October-November 2003 [13,14]. The studied geomagnetic storm was caused by a shock-ICME (interplanetary coronal mass ejection) [15] that first reached the Earth on 11 November 2025, triggering the initial phase of the geomagnetic storm by the end of the day. Following this mentioned tradition, we refer to this event as the “Ugly Duckling Geomagnetic Storm”, named after the Hans Christian Andersen’s world-famous fairy tale, which was first published on 11 November 1843, in Copenhagen, Denmark. Like the fairy-tale duckling, the storm may have initially seemed like an ordinary event. However, it revealed its unique nature: several CMEs merged and impacted the Earth’s magnetosphere extremely, transforming the “Ugly Duckling” storm into a major event that strongly impacted technological systems (communications, radio wave propagation, and navigation optical effects) and produced beautiful auroras visible even at latitudes where polar lights are not usually observed (Figure 1).

This paper analyzes the causes and consequences of the event in near-Earth space, beginning with the Sun as the source of disturbances. It then considers CMEs and their manifestations in solar wind (interplanetary CMEs), Forbush decrease and ground-level enhancement and their impact on the Earth’s magnetic field and the ionosphere, optical airglow and SAR arcs. It also examines the impact on critical technological infrastructures, such as the accuracy of global navigation satellite systems (GNSSs) and radio wave propagation from high-frequency radars.

## 2. Data and Methodology

To study the Sun and the solar wind, we used satellite data. We used images from the Solar Dynamic Observatory (SDO) [16] and the PROBA2 microsatellite mission [17]. The NASA Advanced Composition Explorer (ACE) [18] provided solar wind parameters, including solar wind flow pressure and speed, interplanetary magnetic field, proton density, proton temperature, etc.

We used data from global navigation satellite systems (GNSSs) [19]. GNSSs provide total electron content (TEC) data and TEC-based products. The ROTI (Rate Of TEC Index) is a widely used index for studying small-scale ionospheric irregularities and/or rapid variations in the total electron content (TEC). Based on dual-frequency GNSS measurements, the ROTI index is defined as the standard deviation of the TEC rate over a 5 min time interval [20]:

(1) $$ROTI=\sqrt{\left\langle \left.{\left(\frac{∆I}{∆t}\right)}^{2}\right\rangle \right.-{\left\langle \left.\frac{∆I}{∆t}\right\rangle \right.}^{2}},$$

We used ROTI keograms to study the dynamics of the auroral oval boundary. A keogram is a two-dimensional representation of the time–latitude dependence of the average value. ROTI keograms can be used to observe the onset time and location, zonal width, lifetime, propagation distance, and average velocity of ionospheric irregularities [21]. We selected a ROTI keogram bin size of 2.5° for latitude and 5 min for time.

Global Ionospheric Maps (GIMs) (based on GNSS data) were used to study the global ionospheric dynamics, particularly the equatorial anomaly. GIMs are global regular grids of the TEC in the Earth’s ionosphere. The maps have a spatial resolution of 5° × 2.5° with respect to longitude and latitude and a temporal resolution ranging from 15 min to 2 h. We used two types of GIMs: MosGIM, developed at Moscow State University [22] and implemented in SIMuRG [19], and UQRG GIM, developed by the Universitat Politecnica de Catalunya scientific center [23,24]. MosGIM maps use carrier phase GNSS observations and a single-layer spherical harmonics model, while UQRG maps use a tomographic model and kriging. MosGIM and UQRG GIM have temporal resolutions of 1 h and 15 min, respectively.

GIMs smooth both fast and local processes. To avoid smoothing, we used non-interpolated TEC data—the adjusted TEC. The adjusted TEC is an estimation for the absolute vertical TEC in the ionospheric pierce point, cleaned from geometry and bias effects, and it has high temporal and spatial resolutions [19]. To calculate the adjusted TEC, we use UQRG GIM, performing trilinear interpolation in time and space [19].

**Figure 2:**
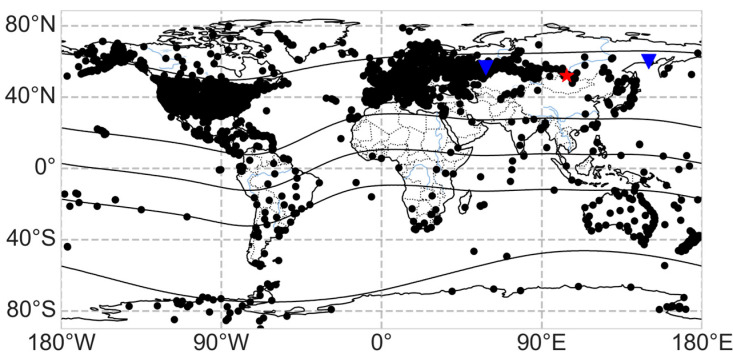
Map of the ground-based facilities. The black dots mark GNSS receivers; the blue triangles mark high-frequency radars; and the red asterisk marks the approximate observation region of the optical complex, the Irkutsk digisonde, and the Sayan Spectrographic Cosmic Ray Complex. The black lines indicate the geomagnetic equator and geomagnetic parallels (±15° MLAT, ±60° MLAT).

We used SIMuRG (https://simurg.space/, accessed on 2 February 2026) [19] to collect and process GNSS data. SIMuRG provides ROTI, TEC variations, and an adjusted vertical TEC. The main data providers (used in this research) are the International GNSS Receiver Network [25], the Federal Center for Navigation Data (https://fcnd.ru/, accessed on 2 February 2026) and the continuously operating reference station network EFT-CORS (https://eft-cors.ru/, accessed on 2 February 2026). Figure 2 shows the locations of GNSS receivers.

To estimate the impact of the storm on GNSS positioning, we calculated the receivers’ coordinates using GPS kinematic Precise Precision Positioning (PPP) based on the GAMP software [26]. The three-dimensional positioning error was calculated as follows:

(2) $$\sigma =\sqrt{{∆X}^{2}+{∆Y}^{2}+{∆Z}^{2}\;},$$

where ∆X, ∆Y, and ∆Z are the deviations of the measured coordinates in three directions from the median coordinates over a period of 23 h (excluding convergence time). We also excluded receivers which the total 3D positioning errors exceeded the threshold of three standard deviations.

The DPS-4 Ionosonde (52° N, 104° E) [27] was used to measure ionospheric plasma parameters (the critical frequencies and heights of the E, F1 and F2 layer maxima). Disturbances represent the deviations in the ionospheric plasma parameter values during the geomagnetic storm on 12 November 2025 compared to a relatively calm day on 11 November 2025. We analyzed the absolute values of the critical frequency ΔfoF2(MHz) and the relative deviations ΔfoF2(%) = 100%·ΔfoF2(MHz)/foF2 for the critical frequency of the F2 layer.

To study cosmic rays, we used the Sayan Spectrographic Cosmic Ray Complex (http://cgm.iszf.irk.ru/, accessed on 2 February 2026) and the cosmic ray station in Norilsk (http://cgm.iszf.irk.ru/nrlk/nrlk.htm, accessed on 2 February 2026) [28]. Both are part of the cosmic ray receiving complex of the Institute of Solar-Terrestrial Physics of Siberian Branch of Russian Academy of Sciences (ISTP SB RAS). The Sayan Spectrographic Cosmic Ray Complex comprises three automatic cosmic ray stations located at different altitudes: 435 m (IRK1, 52.47° N, 104.03° E), 2000 m (IRK2, 51.37° N, 100.55° E) and 3000 m (IRK3, 51.37° N, 100.55° E) above sea level. These stations are equipped with 18-NM-64, 12-NM-64 and 6-NM-64 neutron supermonitors, respectively. The cosmic ray station in Norilsk is equipped with an 18-NM-64 neutron supermonitor. Hourly averaged data are used. The statistical accuracy of observations over the one-hour accumulation period is 0.1%.

To record the upper atmosphere airglow, we used the optical complex located at the ISTP SB RAS Geophysical Observatory (103° E, 52° N) [29]. The all-sky camera records the spatiotemporal variations in the night sky airglow. The camera is directed to the zenith, with an angular field of view of 180°. Automatically replaceable interference filters select the spectral range. We present the data from the 630.0 nm spectral channel (red channel, FWHM 2 nm, the exposure time is 55 s).

We used high-frequency radars deployed by ISTP SB RAS [30]: the EKB (56.5° N, 58.5° E) and the MGW (60° N, 150° E) radars. These radars are SuperDARN-like radars. The radar is a monostatic facility operated at 8–20 MHz, with a field of view of ~50°, width of a single beam of 3–6°, and operating range of 3500–4500 km. The radar’s spatial resolution ranges from 15 to 45 km, and the temporal resolution is ~1–2 min. The radars are part of the national network SECIRA.

## 3. Results

### 3.1. Solar Activity

The main cause of the disturbances in near-Earth space on November 2025 was the generation of a series of geoeffective coronal mass ejections, reflecting the high level of flare activity in the large active region 14274 on the Sun, according to NOAA nomenclature.

The described events occurred during the initial phase of the decline of the 25th solar activity cycle. The monthly average solar activity level of the International Number of Sunspots (version 2.0) [31] was S = 91.7 in November 2025. This is half the maximum value (S = 216) recorded in the current solar cycle on August 2024. The maximum number of sunspots was 154 on 9 November 2025. In November, activity was more prevalent in the southern hemisphere of the Sun, with an average of Ss = 52.2 (compared to Sn = 39.6 in the northern hemisphere).

In November 2025, 33 active regions (AR) were observed on the solar surface, which consisted mainly of small groups of spots with a relatively low level of flare activity. The exception was a large active complex that included AR 14274. This region was observed on the solar surface from 3 to 16 November 2025, with heliocentric (Carrington) coordinates: the latitude of the AR center φ = +24° and the longitude L = 274°, with a passage of the central meridian on 9 November 2025.

**Figure 3:**
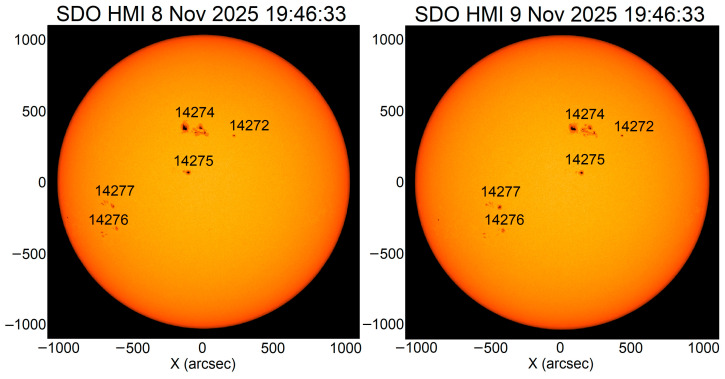
Active regions on the visible solar disk on 8 (**left panel**) and 9 (**right panel**) November 2025 (https://www.solarmonitor.org/, accessed on 2 February 2026) from the SDO HMI data.

**Figure 4:**
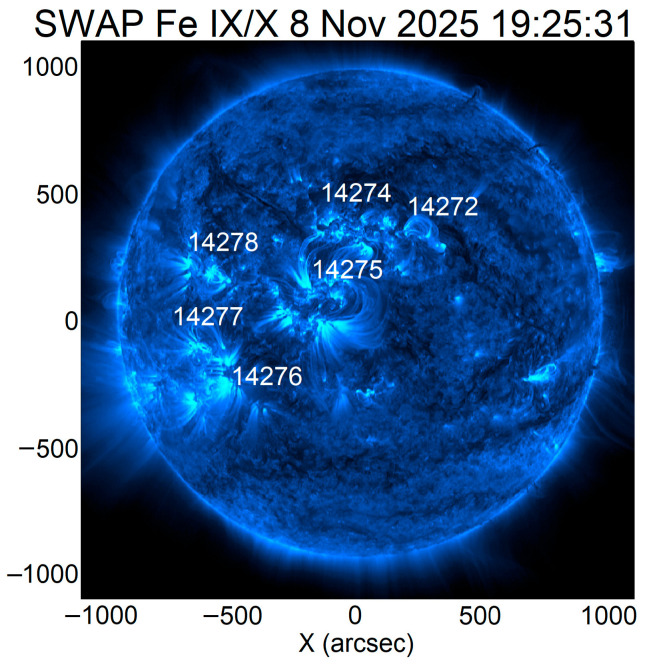
Image of the Sun on 8 November 2025 from PROBA2 SWAP in channel 174 Å.

Figure 3 shows the AR on the visible solar disk on 8 and 9 November 2025. The coordinates of the AR 14274 center were 7° N 6° W at 20:30 UT on 9 November 2025. This active region turned out to be magnetically connected to neighboring AR 14275 in particular. This is confirmed by the loops connecting the two ARs (Figure 4).

The large AR 14274 has reached a maximum area of 1100 millionths of a Solar Hemisphere (ftp://ftp.swpc.noaa.gov/pub/warehouse/2025/, accessed on 2 February 2026). Throughout the entire observation period, the magnetic configuration of this AR corresponded to the beta–gamma–delta class, which is statistically associated with high levels of flare activity. A large number of flares were observed in this AR, including events of the highest X-ray class: 135 C-class flares, 15 M-class flares and 5 X-class flares. Table 1 shows a list of strong flares above M5.0.

On 4 November 2025, solar flare X1.8 occurred. This was the first X-class flare after a five-month timeout, when AR 14274 was located near the eastern limb. Consequently, the coronal mass ejections (CMEs) did not affect the Earth. On 5 November 2025, two M7.45- and M8.6-class flares occurred in AR 14274, accompanied by CMEs. The CME velocity from the second flare reached 1000 km/s. The corresponding interplanetary coronal mass ejection (ICME) reached Earth on 6 November 2025, causing geomagnetic disturbances classified as G2, which lasted three days.

Powerful solar flares of X1.7 and X1.2 class were observed in AR 14274 on 9 and 10 November 2025, respectively. Both flares were accompanied by halo CMEs. According to the type II radio burst data (ftp://ftp.swpc.noaa.gov/pub/indices/events, accessed on 2 February 2026), the speed of the CME-related shock to the X1.7 flare was ~800 km/s, while the velocity of the CME-related shock to the X1.2 flare was 1320 km/s. AR 14274 was in the central zone of the solar disk.

Located in the western part of the solar disk, AR 14274 experienced the strongest solar flares of 2025: X5.1 on 11 November and X4.0 on 14 November. The speed of CME-related shock to the X5.1 solar flare was 1350 km/s (according to type II radio burst data. This CME caused a geomagnetic storm on 13 November 2025 (G3 class according to the Kp index).

On 16–17 November 2025, the active region disappeared behind the western limb of the Sun, continuing to be in a potentially flare-active state.

### 3.2. Solar Wind Conditions

**Figure 5:**
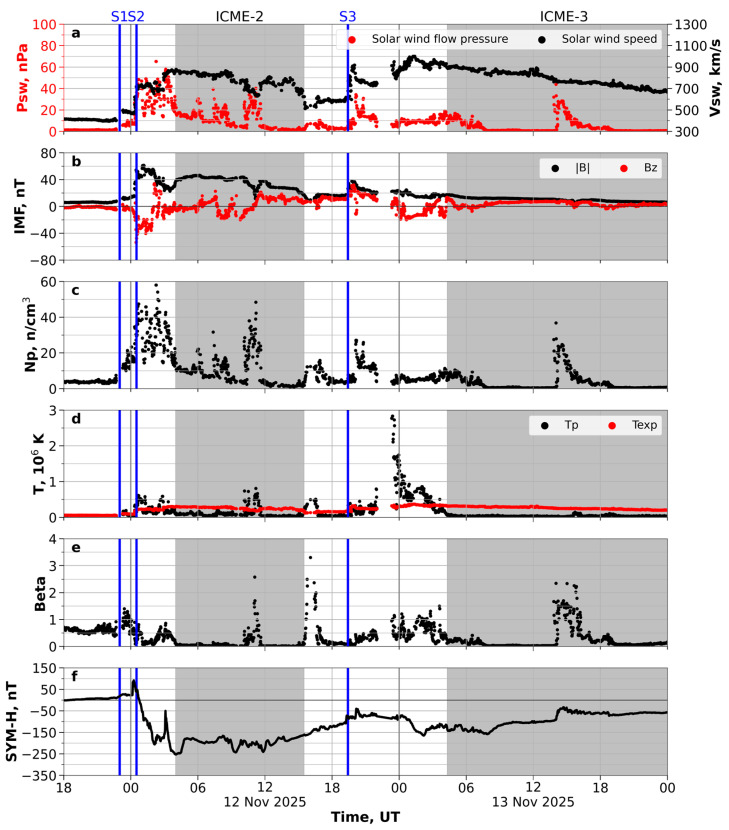
Parameters of the solar wind plasma (**a**), the characteristics of the interplanetary magnetic field (**b**), the proton density (**c**) and temperature (**d**), the plasma beta (**e**), and the SYM-H index (**f**) on 12–13 November 2025. The vertical blue lines S1, S2 and S3 indicate the times of Shock-1, Shock-2 and Shock-3, respectively. The gray regions show the periods of the ICMEs. The data were time-shifted to the Earth’s bow shock nose.

A complex shock-ICME structure produced the Ugly Duckling geomagnetic storm. The ACE spacecraft recorded the shock waves at the L1 Lagrange point at ~22:15 UT on 11 November 2025, 23:48 UT on 11 November 2025 and 18:53 UT on 12 November 2025 (23:15 on 11 November 2025, 00:20 UT on 11 November 2025 and 19:45 UT on 12 November 2025 for data shifted to the Earth’s bow shock nose [33], correspondingly). Figure 5 shows the parameters of the solar wind plasma and the characteristics of the interplanetary magnetic field (IMF) shifted to the Earth’s bow shock nose: the solar wind flow pressure and speed (a) in nPa and km/s, correspondingly; the total field and Bz components of IMF (b) in nT; the proton density Np (c) in n/cm^3^ (where n is the number of protons); the temperature T (d) in K; the plasma beta β (e); and the geomagnetic index SYM-H (f) in nT.

ACE spacecraft recorded the interplanetary shock wave—Shock-1 (Figure 5, vertical line S1)—driven by the 9 November CME at around 22:15 UT on 11 November 2025 (23:15 UT for data shifted to the Earth’s bow shock nose). The velocity jumped at the front of the interplanetary shock wave ranged from ~400 to ~500 km/s, and the IMF increased from 5 to 12 nT. However, this ICME did not feature a large magnetic field and had little influence on the geomagnetic activity. We identified the area behind the Shock-1 wave front as the sheath region because the proton temperature and plasma beta characteristic of the ICME were not abnormally low compared to the background solar wind [34,35,36]. The ICME-1 sheath, the counterpart of this CME, could enter the sheath region behind the second interplanetary shock wave front.

The second interplanetary shock (Shock-2) wave (Figure 5, vertical line S2) arrived at ~23:48 UT on 11 November 2025 (00:20 UT on 12 November 2025 shifting to the Earth’s bow shock nose). The speed of the solar wind at the interplanetary shock wave front increased from ~460 km/s to ~730 km/s, while the Bz component of the interplanetary magnetic field reached −54 nT. Shock-2 propagated into a previous ICME, producing a complex shock-ICME structure [15].

ICME-2 (Figure 5), the driver for Shock-2, hit near-Earth space at around 04:00 UT on 12 November 2025. Inside ICME-2, the proton density decreased up to 10–11 n/cm^3^, the plasma beta dropped much less, 0.5 (during most of the time), and the ratio of the proton temperature to the temperature expectedly [34] lowered below 0.5. ICME-2 lasted around 9 h until 15:30 UT on 12 November 2025. Inside ICME-2, we observed a high-intensity two-hour surge of unknown nature in plasma density and density-dependent characteristics (pressure and plasma beta). During this time period, the proton temperature slightly increased, while the solar wind velocity and the magnetic field slightly decreased. During the registration of the sheath region behind the Shock-2 wave front, the SYM-H index began to decrease and reached its minimum values.

The third interplanetary shock wave (Figure 5, vertical line S3) in the solar wind reached the Earth at the L1 point at 18:53 UT on 12 November 2025 (after ~19:45 UT at the Earth’s bow shock nose). The solar wind speed increased to ~920 km/s, the total IMF increased from ~20 nT to 38 nT, and the southern component of the IMF Bz reached about −14 nT. ICME-3, the driver of Shock-3, started at ~04:00 UT on 13 November 2025 and continued until the end of the day. Within this ICME, we also observed a high-intensity surge of unknown nature in plasma density, pressure, and plasma beta, which lasted for about five hours.

### 3.3. Geomagnetic Conditions

**Figure 6:**
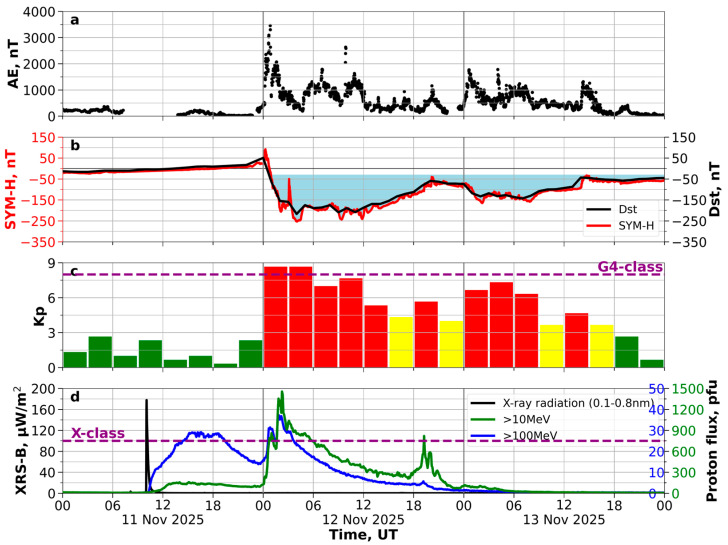
AE (**a**), Dst and SYM-H (**b**), and Kp (**c**) indices and the soft X-ray emission (black) and proton flux with energies exceeding 10 MeV (green) and 100 MeV (blue) (**d**) on 11–13 November 2025. In (**c**), green indicates a Kp index of 0–3, yellow indicates a Kp index of 4–6, and red indicates a Kp index of 7–9.

The geomagnetic activity indices show changes in the Earth’s magnetic field caused by solar wind plasma. They also display variations within the magnetosphere and its interactions with the ionosphere. Figure 6 shows the AE, Dst, SYM-H and Kp indices, the soft X-ray emission and the proton flux (in proton flux unit (pfu) = 1 proton/(cm^2^ s sr)) with energies exceeding 10 MeV and 100 MeV.

The Kp index is one of the planetary indices, calculated by averaging data over a three-hour period. It indicates global changes in the Earth’s magnetic field. It is calculated as a weighted average of the indices from 13 magnetic observatories located in the mid-latitudes [37]. The Dst index is used as a measure of the magnetic field caused by ring currents that occur during geomagnetic storms in the Earth’s magnetosphere [38]. It is determined based on data from observatories located at low latitudes and evenly distributed in longitude. At the Earth’s surface, the influence of ring currents is expressed as a decrease in the horizontal component of the magnetic field. In contrast to the Dst index, the geomagnetic index SYM-H is calculated using data from a larger number of observatories and has a one-minute resolution [39]. Essentially, SYM-H is the “minute-by-minute” version of the Dst index, and thus both indices (SYM-H and Dst) show an identical picture of storm development; the SYM-H index is more detailed.

The geomagnetic storm on 12 November 2025 developed according to the classical three-phase scenario. The initial phase began on the night of 11–12 November 2025 and was characterized by positive increases in the Dst and SYM-H indices (Figure 6b). Before the shock waves, Bz (Figure 5b) was slightly negative (mainly −1.5 nT, but reaching −5 nT). After 19:30 UT on 11 November 2025, the Dst and SYM-H indices increased to ~+10 nT. At around 23:00 UT, the SYM-H and Dst indices increased sharply to +17 nT and +27 nT, respectively, accompanied by an increase in auroral activity (the AE index). Then, SYM-H increased to +92 nT at 00:16 UT on 12 November 2025. These changes indicated the beginning of global disturbances. The Dst index then sharply decreased to a minimum of −217 nT at around 05:00 UT on 12 November 2025, reflecting the intensification of the ring current. These changes made this storm the third strongest in solar cycle 25 after the extreme events of May 2024 (−406 nT) and October 2024 (−333 nT). The Kp index, reflecting the intensity of convective processes, reached its maximum level of 9-, clearly correlating with an increase in the solar wind speed, a sharp decrease in the negative value of the Bz component of the interplanetary magnetic field, and a decrease in the Dst index. A sharp increase in the solar wind plasma density indicated a shock wave.

When Bz is negative, magnetic reconnection occurs at the daytime magnetopause, leading to the extension of the polar cap and the equatorward displacement of the auroral oval. During the main phase of the geomagnetic storm, a relative stabilization was observed at low Dst values. During this time, the Kp index remained high, indicating constant fluctuations in the magnetic field at different latitudes. Another sharp increase in geomagnetic disturbance was recorded at ~10:00 UT on 12 November 2025, which led to a drop in the Dst index to −209 nT (Figure 6b) and an increase in the Kp index to 7+ (Figure 6c). Increased fluctuations in the interplanetary magnetic field and an increase in the speed and pressure of the solar wind were observed (Figure 5a,b).

The recovery phase began with the rotation of the Bz component of the interplanetary magnetic field to the north (Figure 5b). The recovery rate was classic: a relatively rapid increase in the Dst index in the first 6–8 h, followed by a smoother one. It took more than two days to fully recover. The Kp index declined much faster. At 4–6 h after the Bz rotation, the Kp dropped to the level of 4–5, and a day later returned to the standard values of 1–3, which are typical for a geomagnetically calm environment.

The fall and subsequent increase in the Dst, as well as the variable high Kp values during the day, reflect the typical evolution of a geomagnetic disturbance, characterized by rapid changes and significant deviations from calm conditions.

Based on GOES-18 data (Figure 6d), the flux of protons with energies of ≥10 MeV reached ~1450 pfu at 02:15 UT on 12 November 2025. Then, it decreased to ~200 pfu before rising again to ~820 pfu at 19:15 UT on 12 November. The proton flux with an energy of ≥100 MeV exhibited two peaks, reaching peak values of ~30 pfu at 15:30 UT on 11 November and ~37 pfu at 02:10 UT on 12 November.

### 3.4. Cosmic Ray Intensity: Forbush Decrease and Ground-Level Enhancement

Since the beginning of November 2025, a change in the parameters of the solar wind has been observed (Figure 5a). The geomagnetic storm that occurred on 6 November 2025 (the Bz component varied from −10 to −20 nT, the minimum Dst index was −129 nT at 07:00 UT) was accompanied by a decrease in the cosmic ray intensity, called the Forbush effect. The greatest decrease in cosmic ray intensity (compared to the undisturbed period of 29 October) was observed on 7 November: −11.6% at the IRK1 station at 16:00 UT, −11.8% at the IRK2 station at 16:00 UT, −12.1% at the IRK3 station at 16:00 UT, and −8.6% at the Norilsk station at 17:00 UT.

**Figure 7:**
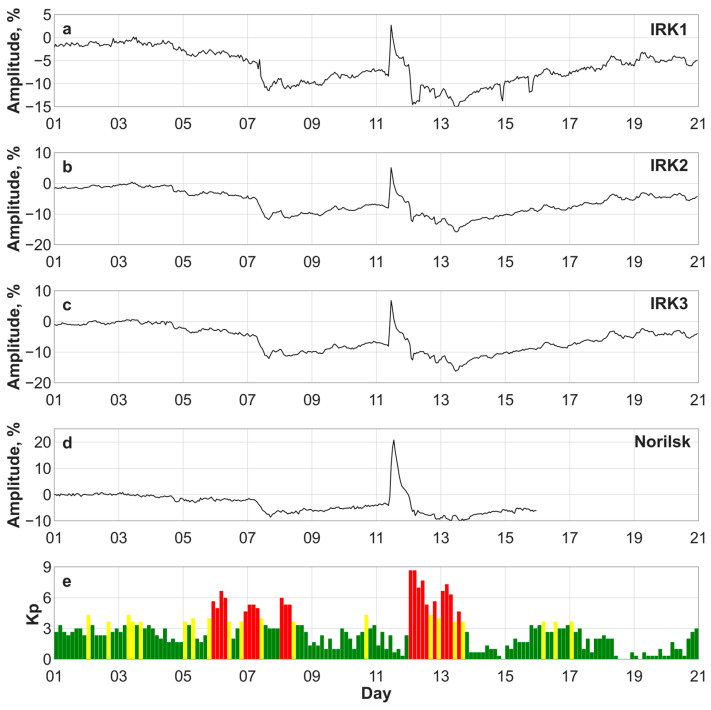
Cosmic ray intensity at the mid-latitude cosmic ray stations of the Sayan Spectrographic Cosmic Ray Complex IRK1 (**a**), IRK2 (**b**), and IRK3 (**c**), and at the high-latitude station in Norilsk (**d**), and the Kp index (**e**) in November 2025. In (**e**), green indicates a Kp index of 0–3, yellow indicates a Kp index of 4–6, and red indicates a Kp index of 7–9.

The cosmic ray level did not recover to the background levels (Figure 7) by 11–13 November: the decrease was −5...−10% relative to the background. On 12 November 2025, after the arrival of the first and second shock waves and the ICME, the intensity of cosmic rays decreased further during the geomagnetic storm. On 13 November, the cosmic ray intensity was −15.0% at the IRK1 station, −15.8% at the IRK2 and IRK3 stations, and −9.9% at the Norilsk station.

Between the two Forbush decreases in cosmic rays on 6–7 and 11–13 November 2025, the cosmic ray intensity increased on 11 November (Figure 7). The ground-level enhancement (GLE) in cosmic ray intensity is associated with the X5.1 solar flare on 11 November 2025 (Table 1) in active region 4274 (with coordinates 24° N 36° W). This event is recorded in the GLE database as event 77 (http://gle.oulu.fi, accessed on 2 February 2026). GLE77 began at ~10:15 UT on 11 November 2025. The maximum amplitude of the increase in cosmic ray intensity was observed at 13:00 UT on 11 November 2025: 24.9% at the high-latitude cosmic ray station in Norilsk and 8.8%, 13.3%, and 14.9% at the mid-latitude cosmic ray stations of the Sayan Spectrographic Cosmic Ray Complex (IRK1, IRK2 and IRK3, respectively).

### 3.5. Ionospheric and Atmospheric Response to the Geomagnetic Storm

#### 3.5.1. Global Ionospheric Structures

##### ROTI Data for Small-Scale Ionospheric Irregularities

**Figure 8:**
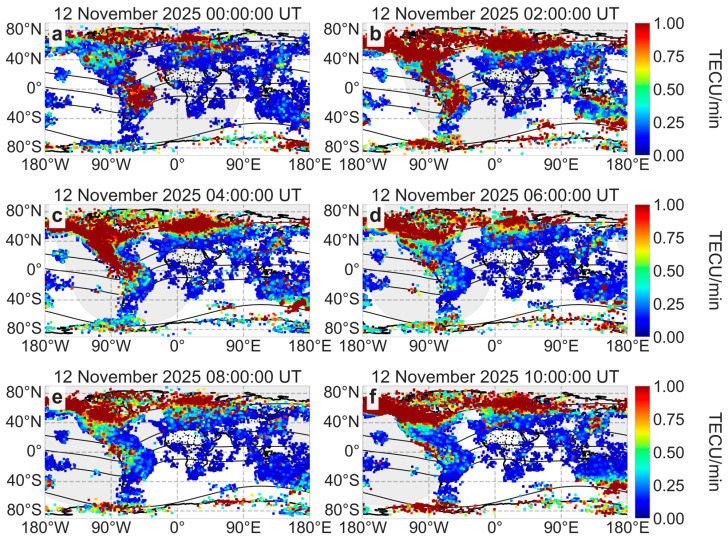
ROTI maps during the main phase of the geomagnetic storm on 12 November 2025 at 00:00 UT (**a**), 02:00 UT (**b**), 04:00 UT (**c**) and during recovery phase at 06:00 UT (**d**), 08:00 UT (**e**) and 10:00 UT (**f**). The black lines indicate the geomagnetic equator and geomagnetic parallels (±15° MLAT, ±60° MLAT).

The ROTI index is widely used to characterize the irregular structure of the ionosphere depending on the geographical latitude and longitude during geomagnetic storms [20]. Figure 8 shows the global ROTI dynamics on 12 November 2025. The full ROTI dynamics on 11–13 November 2025 (with a time resolution of 30 s) can be viewed on SIMuRG (https://simurg.iszf.irk.ru/result?id=691a8a2e35bc61a051b75203, accessed on 2 February 2026).

Since the beginning of the main phase of the geomagnetic storm, the auroral oval considerably intensified up to 1.5 TECU/min. Individual values of the ROTI increased up to 2 TECU/min. The auroral oval boundaries expanded equatorward, crossing the latitudes of 35° N in the American sector and 45° N in the European sector. The auroral oval reached its maximum expansion at 04:00 UT on 12 November 2025. Increased ROTI values (more than 1 TECU/min) near the equator in the American sector indicate the displacement of the equatorial plasma bubble. Since the beginning of the main phase of the geomagnetic storm, the bubble has moved in a northwesterly direction toward the auroral oval boundaries, but the two ionospheric structures did not intersect. They were constantly separated by a band of reduced ROTI values (less than 0.2 TECU/min).

At the beginning of the recovery phase of the geomagnetic storm, at 05:00 UT on 12 November 2025, the auroral oval gradually weakened and its boundaries slowly “returned” to their normal position. However, since 09:00 UT on 12 November 2025, the auroral oval boundaries have been expanding equatorward again. This behavior is related to the dynamics of the Dst index: at 10:00 UT on 12 November 2025, the Dst repeatedly reduced to −209 nT, which was 8 nT higher than the minimum Dst value. After 11:30 UT, the intensity of the auroral oval decreased, and its boundaries shifted poleward.

**Figure 9:**
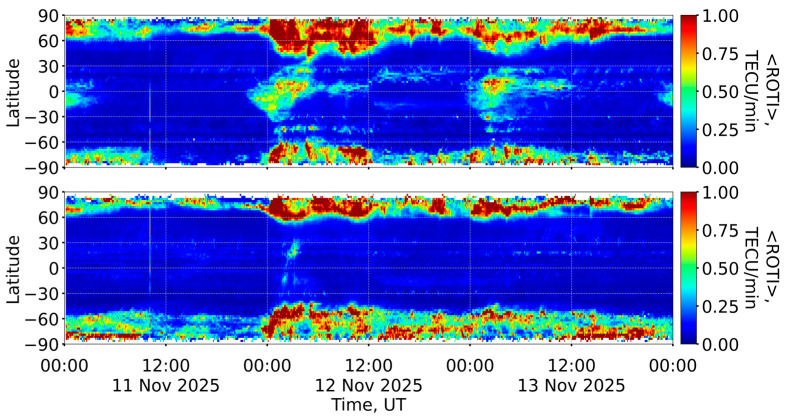
ROTI keograms in the American sector (**top panel**) and the European–Asian sector (**bottom panel**) during 11–13 November 2025.

Figure 9 shows the ROTI keograms in the American and European–Asian sectors.

On 11 November 2025, there were no significant variations in the ROTI at high latitudes. The auroral oval boundaries did not cross 60° in either hemisphere, indicating calm conditions (Figure 9). From the beginning of the main phase of the geomagnetic storm, the auroral oval boundaries shifted equatorward. According to the ROTI keogram, the maximum displacement of the auroral oval boundaries occurred between 04:00 UT and 05:00 UT on 12 November 2025. The auroral oval boundaries reached 30–35° N and 60° S in the American sector and 45–50° N and 50° S in the European–Asian sector. An increase and poleward shift in the auroral oval boundary were observed until about 12:00 UT on 12 November 2025 (the beginning of the recovery phase). This correlated with the geomagnetic conditions and the lowered Dst values (Figure 6b).

On the ROTI keogram (Figure 9, top panel) the motion of the equatorial plasma bubble can be clearly observed. From the beginning of the geomagnetic storm, the bubble shifted northwards to about 30° N. Despite the counter propagation of the auroral oval and the equatorial plasma bubble, these two structures did not cross: a band of low ROTI values separated them at a latitude of 30° N.

##### Global Ionospheric Maps of Total Electron Content

**Figure 10:**
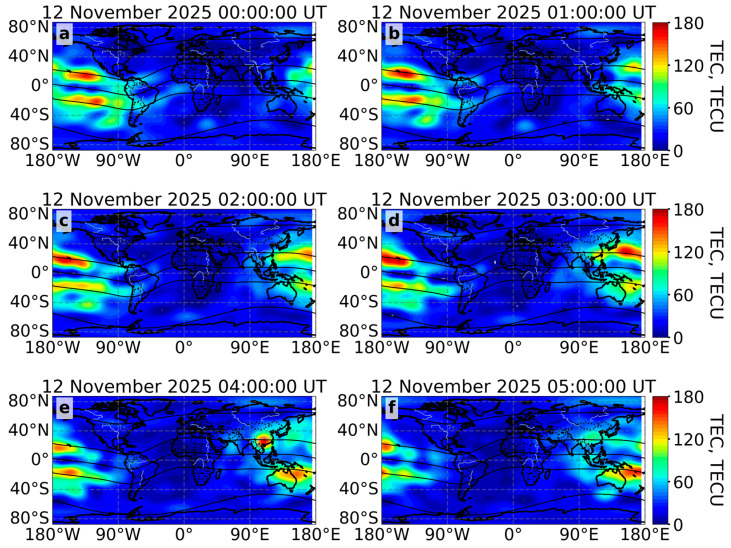
Global Ionospheric Maps (MosGIM) during the main phase of the geomagnetic storm on 12 November 2025 at 00:00 UT (**a**), 01:00 UT (**b**), 02:00 UT (**c**), 03:00 UT (**d**), 04:00 UT (**e**) and during recovery phase at 05:00 UT (**f**). The black lines indicate the geomagnetic equator and geomagnetic parallels (±15° MLAT, ±60° MLAT).

The equatorial anomaly is a feature of the equatorial ionosphere which can be characterized by Global Ionospheric Maps [22] (Figure 10).

During the main phase of the geomagnetic storm, the equatorial anomaly crests intensified and shifted poleward. The maximum intensification and shift occurred from 03:00 UT to 04:00 UT on 12 November 2025, when TEC values reached 150–175 TECU. The equatorial anomaly crests were observed at about 30° N in the northern hemisphere and 25° S in the southern hemisphere. At the end of the main phase (from 04:00 UT to 05:00 UT), the “northern” equatorial anomaly crest weakened sharply, dropping from 150 TECU to 80–90 TECU. Conversely, the “southern” equatorial anomaly crest remained at high values of 140–150 TECU (Figure 10f).

**Figure 11:**
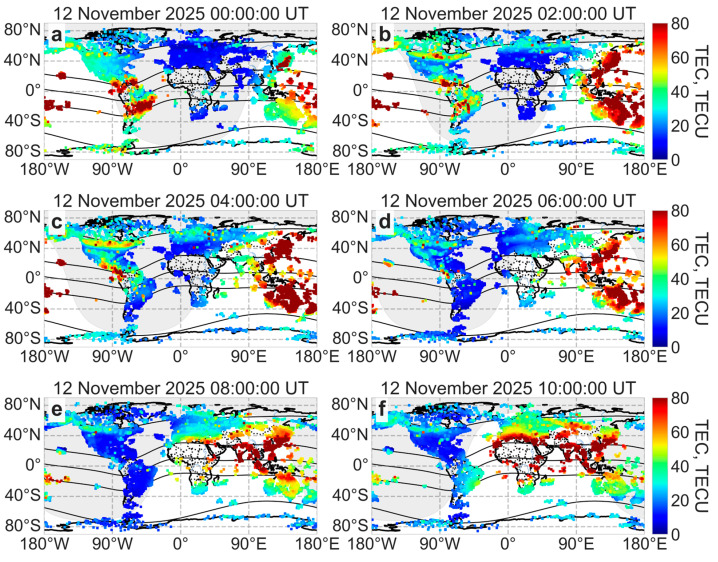
Maps of adjusted TEC during the main phase of the geomagnetic storm on 12 November 2025 at 00:00 UT (**a**), 02:00 UT (**b**), 04:00 UT (**c**) and during recovery phase at 06:00 UT (**d**), 08:00 UT (**e**) and 10:00 UT (**f**). The black lines indicate the geomagnetic equator and geomagnetic parallels (±15° MLAT, ±60° MLAT).

Figure 11 shows global ionization during the 12 November 2025 geomagnetic storm.

During the main phase of the geomagnetic storm, an area of increased ionization was observed at the auroral oval boundary, reaching latitudes of 40–45° N in the American sector and 50–55° N in the European–Asian sector. The TEC increased to 60–70 TECU in the western hemisphere and to 40–50 TECU in the eastern hemisphere. The area of increased ionization gradually decreased by 09:00 UT on 12 November 2025. Between 09:00 UT and 10:00 UT, the area of increased ionization near the auroral oval boundaries increased again. After 10:00 UT, ionization began to weaken. In the southern hemisphere, the response to the geomagnetic storm was weaker, with the TEC reaching approximately 40 TECU and increased ionization occurring at latitudes of about 60–70° S. The dynamics of ionization at high latitudes are consistent with the dynamics of the auroral oval.

The equatorial plasma bubble moved northwest towards the auroral oval in the American sector (increased ionization areas of 60–100 TECU). However, the two regions did not intersect, as the reduced ionization of about 20 TECU separated the two structures (Figure 11). This is consistent with the ROTI maps (Figure 8).

#### 3.5.2. Mid-Latitude Ionosphere (Over Irkutsk)

**Figure 12:**
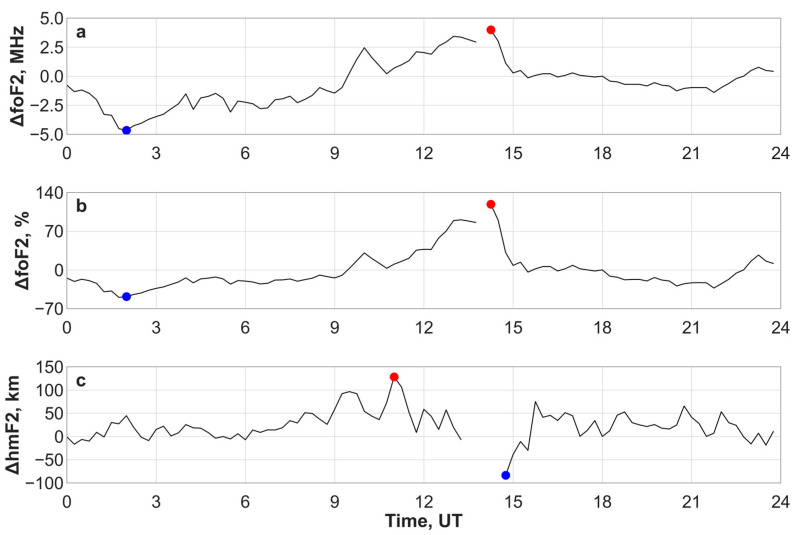
Ionospheric disturbance over Irkutsk (mid-latitude Asian sector, 52.2° N, 104.4° E) on 12 November 2025: ΔfoF2(MHz) (**a**), ΔfoF2(%) (**b**) and ΔhmF2(km) (**c**). The red and blue points indicate the maximum and minimum values of the parameters, respectively.

To study the mid-latitude ionospheric response to the 12 November 2025 geomagnetic storm, we used the Irkutsk ionosonde [27] located in a mid-latitude region of Asia. We analyzed the critical frequencies and heights of the E, F1 and F2 layer maxima and compared them with the parameters of a relatively calm day (we chose 11 November 2025). A comparison of these parameters showed that the geomagnetic storm had the greatest impact on the F2 layer. Therefore, we further analyzed the disturbances of the critical frequency (ΔfoF2) and maximum height (ΔhmF2) of the F2 layer. Figure 12 shows the variations in ΔfoF2(MHz), ΔfoF2(%) and ΔhmF2(km) on 12 November 2025.

At the beginning of the main phase of the geomagnetic storm, we observed a negative foF2 disturbance. At around 02:00 UT (09:00 LT) on 12 November 2025, the disturbance reached extreme negative values: ΔfoF2 = −4.7 MHz, ΔfoF2 = −50%. The foF2 decreased by more than twice, and the NmF2 decreased by more than four times. This decrease in foF2 and NmF2 can be explained by the transfer of molecular nitrogen from high to mid-latitudes, caused by the rapid increase in geomagnetic activity (Figure 6).

During the recovery phase, the negative foF2 disturbance turned into a positive one and lasted for 6 h, from 09:30 UT to 15:15 UT (16:30–22:15 LT), covering the local evening and night times. At 14:15 UT (21:15 LT) on 12 November 2025, the foF2 increased by more than twice (ΔfoF2 = +4 MHz, ΔfoF2 = +119%) and NmF2 increased by more than four times (ΔNmF2 > +300%). During the positive foF2 disturbances, we observed both extremely positive and extremely negative values of ΔhmF2: ΔhmF2 = +128 km at 11:00 UT (18:00 LT) and ΔhmF2 = −83 km at 14:45 UT (21:45 LT).

#### 3.5.3. Optical Effects

**Figure 13:**
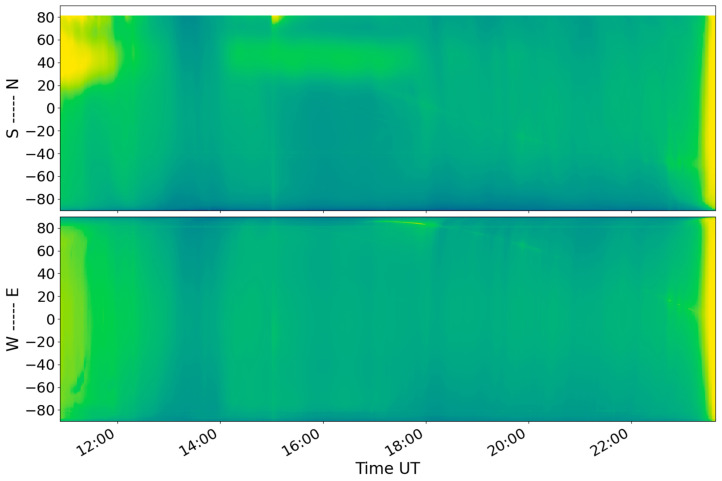
Keogram of the OI 630 nm airglow intensity from the ASI0 camera on 12 November 2025. The direction is north–south (**top panel**) and east–west (**bottom panel**).

On 12 November 2025 in the Asia sector, the ISTP SB RAS optical complex [29] recorded mid-latitude auroras in the OI 630 nm airglow, but recorded no effects in the other major atmospheric airglows. The all-sky cameras recorded an SAR arc from the beginning of the optical measurements at 10:50 UT until ~13:00 UT and again from ~14:00 UT until ~18:00 UT (Figure 13). From 13:00 UT to 14:00 UT, the SAR arc was not observed behind continuous clouds.

The apparent width of the SAR arc changed from ~40 degrees at 10:50 UT to ~20 degrees between ~14:00 UT and ~18:00 UT (Figure 13), with the midpoint positioned at ~40 degrees from the zenith.

SAR arcs—a type of aurora observed at subauroral and mid-latitudes during intense geomagnetic storms—exhibit a pronounced spatial structure [40]. They manifest as spatially extended, diffuse red (630.0 nm) airglow in the upper atmosphere, which is more intense against the background emission. In some classifications, SAR arcs are considered a type “d” of mid-latitude aurora [40]. The most intense type “d” mid-latitude auroras are observed during the main phases of geomagnetic storms, but SAR arcs are observed both in the recovery phase and in the main phases of geomagnetic storms [41].

**Figure 14:**
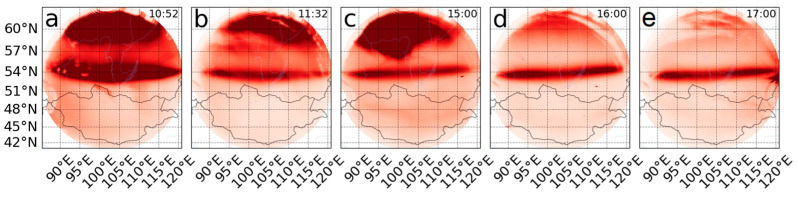
ASI0 all-sky camera snapshots for 10:52 UT (**a**), 11:32 UT (**b**), 15:00 UT (**c**), 16:00 UT (**d**), and 17:00 UT (**e**). Data were mapped for an altitude of 300 km.

Figure 14 shows ASI0 camera snapshots for 10:52 UT (a), 11:32 UT (b), 15:00 UT (c), 16:00 UT (d), and 17:00 UT (f). The frames were mapped for an altitude of 300 km. An increase in aurora on the northern horizon in the 630 nm line was observed at the beginning of the measurements from 10:50 UT to 11:40 UT (Figure 14a,b) and from 15:00 UT to 15:20 UT (Figure 14c).

### 3.6. GPS Positioning Errors

**Figure 15:**
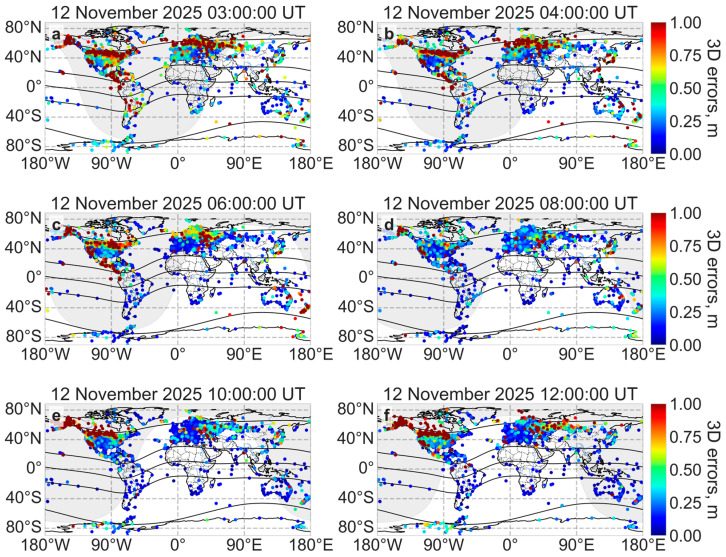
Positioning errors during the main phase of the geomagnetic storm on 12 November 2025 at 03:00 UT (**a**), 04:00 UT (**b**) and during recovery phase at 06:00 UT (**c**), 08:00 UT (**d**), 10:00 UT (**e**) and 12:00 UT (**f**). The black lines indicate the geomagnetic equator and geomagnetic parallels (±15° MLAT, ±60° MLAT).

To evaluate the accuracy of the GPS positioning, the total positioning errors were calculated using Equation (2) in kinematic PPP mode using the GAMP software [26]. A feature of the GAMP software is PPP convergence at the beginning of the day. So, in the first two hours, the increased values of the positioning errors were unrelated to geomagnetic conditions. Figure 15 shows the global dynamics of positioning errors during the second half of the main phase of the geomagnetic storm (from 02:00 UT to 05:00 UT on 12 November 2025).

After PPP convergence at about 02:00 UT on 12 November 2025, positioning errors increased in both the American and European–Asian sectors at high latitudes (around 60° N geomagnetic latitude). In these regions, the positioning errors exceeded 1 m, reaching 2–3 m in some receivers. From 02:00 UT to 04:00 UT, these increased positioning errors gradually shifted towards the mid-latitudes (down to 40–45° N). This displacement correlated to the expansion of the auroral oval boundaries (Figure 8) and the increased ionization (Figure 11). Increased positioning errors were also observed near the equator in the American sector, which correlated with the movement of the equatorial plasma bubble in this region. After 06:00 UT on 12 November 2025, the positioning errors decreased and returned to “normal” values for undisturbed conditions. The average value did not exceed 0.2–0.3 m.

At 09:00 UT on 12 November 2025, the positioning errors sharply increased to 2 m at high latitudes in North America (around 60° N geomagnetic latitude). At this time, ionizations increased (Figure 11) and the auroral oval boundaries expanded (Figure 8) at high latitudes in North America. Until 12:30 UT on 12 November 2025, increased positioning errors were observed at high latitudes in North America. During this period, the Dst index did not exceed −175 nT (Figure 6b). After 12:30 UT on 12 November 2025, the positioning errors decreased to 0.3 m over a period of 1.5 h.

### 3.7. High-Frequency Radio Wave Propagation

**Figure 16:**
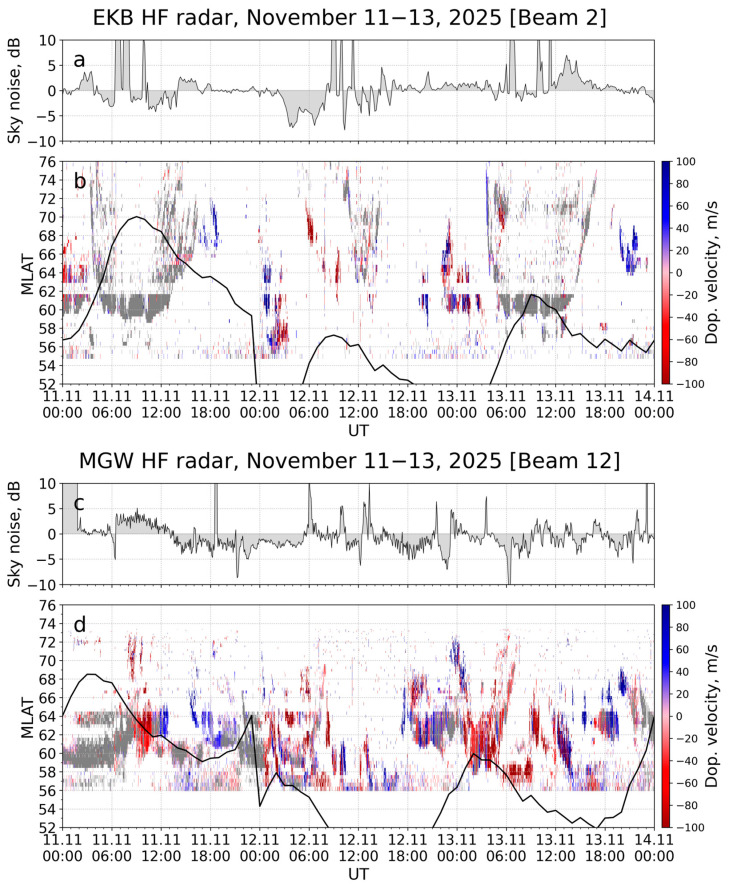
Doppler velocity (against geomagnetic latitude and time) recorded by coherent high-frequency radars EKB (**b**) and MGW (**d**) and the radar sky noise measurements (**a**,**c**). Gray shading denotes ground scatter; colored areas represent ionospheric scatter. The black lines show the position of the bottom of the main ionospheric trough as estimated by [42].

Coherent high-frequency [30] CUTLASS-type radars located in the mid-latitude of Asia were used in this study. Figure 16 shows the measured Doppler velocity from the EKB (56.5° N, 58.5° E; panel b) and the MGW (60° N, 150° E; panel d) radars for the beams closest in direction to the north. Gray shading denotes ground scatter, while colored areas represent ionospheric scatter. Panels a and c of Figure 16 display radar sky noise measurements, expressed as deviations from their monthly medians. Enhanced absorption, a clear signature of the main phase, is evident in the EKB data. During the recovery phase, intervals of enhanced absorption alternated with periods of increased radio noise.

During the main phase of the geomagnetic storm, both radars detected intense ionospheric scattering at relatively close ranges (200–800 km). This likely indicates the southward expansion of the auroral oval and scattering from its structures. The MGW data (12–13 November 2025, 00–07 UT) show a classic pattern: ionospheric scatter first approached the radar (decreasing range) and then receded from it (increasing range). Furthermore, the EKB radar recorded the disappearance of regular ground scatter on 12 November 2025, while the MGW radar exhibited this absence over two days (12–13 November 2025). The black line in Figure 16 shows the position of the bottom of the main ionospheric trough (MIT) as calculated by the model [42] based on the dynamics of the Ap index in the corresponding longitude sectors. Ionospheric scattering was observed either to the north or directly near the bottom of the MIT for both radars. At the beginning of the main phase (00–03 UT 12 November 2025), the main ionospheric trough reached 51° N (47° MLAT) in the EKB radar, and during the recovery phase (00–03 UT 13 November 12) it reached 53° N (49° MLAT). In the MGW radar region, during the period 11–20 UT 12 November 2025, the MIT was located at about 55° N (49° MLAT). At the longitude of Irkutsk (104° E), the MIT reached a minimum latitude of 54° N (49° MLAT) at 00–01 UT and 19–20 UT on 12 November 2025. These time periods preceded the greatest decrease in the critical frequency and a slight increase in the maximum height of the F2 layer according to the ionosonde data (Figure 12).

### 3.8. Blackouts in High-Frequency Radio Sounding Data

**Figure 17:**
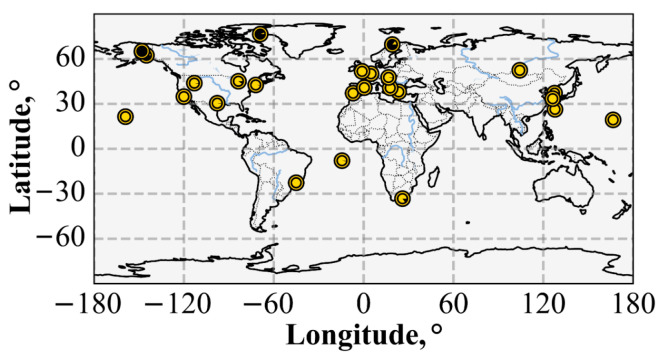
Blackouts during the 12 November 2025 geomagnetic storm, as monitored by the GIRO digisonde network. The data covered period from 03:00 UT on 12 November 2025 to 11:00 UT on 13 November 2025. The black fill in the circles shows the proportion of time during which the digisondes did not record any useful signal.

To study the impact of geomagnetic storms on high-frequency radio signals, we recorded blackouts during the 12 November 2025 geomagnetic storm, as monitored by the GIRO digisonde network (https://giro.uml.edu/, accessed on 23 February 2026). A blackout is an event when a digisonde does not record any useful signal. We analyzed data from 26 stations of vertical sounding from 03:00 UT on 12 November 2025 to 11:00 UT on 13 November 2025, when the Dst index dropped below −100 nT. At five high-latitude stations (Eielson, Gakona, Poker Flat, Thule, and Tromsø), blackout appeared during more than 80% of the soundings. At two mid-latitude stations (Alpena and Grahamstown), blackouts appeared during ~10% of the soundings. Other stations featured ~3% or fewer blackouts. In addition to blackouts, the mid-latitude ionosonde data featured F-spread, Z-mode, and oblique (off-vertical) echoes. Figure 17 shows blackouts from 26 stations of vertical sounding during the 12 November 2025 geomagnetic storm.

## 4. Discussion

Solar activity significantly impacts the Earth. A high level of flare activity was observed in November 2025. On 12 November 2025, a severe G4 geomagnetic storm (Dst = −217 nT) occurred. This event was the third most intense storm of solar cycle 25, after the extreme geomagnetic storm in May 2024 (Dst = −406 nT) [43] and the October 2024 severe storm (Dst = −333 nT) [44]. The geomagnetic indices during the 12 November 2025 storm resemble ones during the famous 2015 St. Patrick’s Day superstorm (Dst = −223 nT), the most powerful storm of solar cycle 24 [45].

Shen et al. [15] discovered that, during multiple CME events, the penetration of a shock into a previous ICME can produce a complex shock-ICME structure. These structures can enhance the CME’s influence on the magnetosphere and double the magnetic storm intensity. They observed the shock-ICME structure as a result of more separated CMEs compared to the November 2025 event. Therefore, we hypothesize that the shock-ICME appeared on 12 November 2025 and significantly increased the intensity of the magnetic storm.

Shen et al. [15] also indicated that, unlike a typical ICME, the proton flux intensity in a shock-ICME increases at the front edge and decreases at the trailing edge. Figure 6d shows an increase in proton flux (both >10 MeV and >100 MeV) after Shock-2, providing additional evidence for a shock-ICME structure.

We studied the global and local effects of the 12 November 2025 geomagnetic storm in near-Earth space based on multi-instrument data, including GNSS, the DPS-4 ionosonde, the cosmic ray receiver complex, high-frequency radars, and the ACE solar wind satellite. At the beginning of the main phase of the geomagnetic storm (from 01:00 UT to 02:00 UT on 12 November 2025), the Dst index rapidly dropped from +51 nT to −65 nT (the fall rate was 116 nT per hour), while, during the May 2024 extreme geomagnetic storm [46], the index dropped from +61 nT to −36 nT (97 nT per hour), and, during the October 2003 storm [14], the index dropped from −10 nT to −105 nT (95 nT per hour). The main phase was short-lived, occurring from 01:00 UT to 05:00 UT on 12 November 2025, with a total Dst drop of 268 nT (from +51 nT to −217 nT). These rapid changes disturbed the magnetosphere and ionosphere.

During a geomagnetic storm, the intensity of small-scale irregularities increases and plasma instabilities occur [47]. Sharp ionospheric density gradients form [48], and TEC amplitude jumps are observed, particularly at high latitudes [7]. A complete signal failure [49] and GNSS positioning failures [7] occur. The positive correlation between an increase in the phase failure density and the intensity of TEC variations during geomagnetic storms suggests that an increase in measurement failures may be associated with ionospheric plasma disturbances [50].

During the main phase of the 12 November 2025 geomagnetic storm, the ROTI index increased up to 1.5 TECU/min (Figure 8), and ionizations increased up to 60–70 TECU (Figure 11) at high latitudes. The auroral oval boundaries, characterized by increased ROTI and ionization, expanded to 35° N in the American sector and 50° N in the European sector. The expansion of auroral oval boundaries was also observed during other geomagnetic storms: however, the intensification of ROTI and the maximum expansion of the auroral oval boundaries depend on the intensity of the geomagnetic storm [51,52,53]. During a similar geomagnetic storm in solar cycle 25 (10–11 October 2024, Dst = −333 nT, Kp = 9-), the ROTI index reached 1.5–2 TECU/min, and the auroral oval boundaries expanded to 35° N in the American sector and 50° N in the European sector. During the extreme geomagnetic storm (10–11 May 2024, Dst = −406 nT, Kp = 9), the auroral oval boundary expanded more strongly, reaching 30° N in the American sector and 45° N in the European sector.

The mid-latitude ionosphere in the Asian sector (52° N, 104° E) featured a positive foF2 disturbance during the recovery phase on 12 November 2025. The foF2 increased by more than twice (ΔfoF2 = +119%), and NmF2 increased by more than four times (ΔNmF2 > +300%). Such large positive NmF2 disturbances have not been observed before. The positive phase lasted almost 6 h, covering the evening and night local times. The height of the hmF2 maximum experienced both extremely positive and extremely negative disturbances (Figure 12c). This is not the classic dusk effect (plasma elevation) because during the dusk effect the hmF2 disturbance would only be positive, and the positive foF2 disturbance would be shorter. Thus, the dusk effect can only be an integral part of the process. Horizontal plasma transfer could be an alternative reason for this disturbance, either from the dayside to the nightside (from west to east), from southern to northern latitudes, or their combination. Another possible reason is an expansion of the equatorial anomaly crests to the Irkutsk mid-latitudes, but we did not observe this (Figure 8).

An equatorial plasma bubble is a typical ionospheric disturbance observed in the evenings and at midnight near the equator [54]. Tulasi Ram et al. [55] observed an equatorial plasma bubble before sunrise during geomagnetic activity, but this is a rare occurrence. During geomagnetic storms, the equatorial plasma bubble can move to higher latitudes [56,57]. During the severe geomagnetic storm on 12 November 2025, we observed an equatorial plasma bubble moving northwest towards the auroral oval in North America (Figure 8c). The two ionospheric structures did not intersect: a band of low ROTI values (Figure 8) or low ionization (Figure 11c) separated them. A similar poleward movement of an equatorial plasma bubble was observed during the geomagnetic storm on 10–11 October 2024 [58]. During that storm, the equatorial plasma bubble rapidly extended poleward, reaching 30° N in North America, and occasionally came very close to the equatorward-expanded oval.

Pulinets et al. [59] also recorded the formation of a giant plasma bubble during this storm, based on LAERT equipment on board the Ionosphere-M satellite [60]. They found that the bubble reached an altitude of 500 km. Combining these findings with the spatial characteristics obtained above, we could insist that it was a super bubble event, more intense than the 2006 super bubble [57] and comparable to the May 2024 event [61].

A geomagnetic storm affects the equatorial ionosphere [62,63], especially the equatorial ionization anomaly [64]. The prompt penetration electric field (penetrated magnetospheric/solar wind electric fields) [65] and the disturbance dynamo electric field (due to disturbed thermospheric winds) [66] change the equatorial zonal electric field, influencing the equatorial plasma fountain and equatorial plasma bubble [67]. The intensified fountain effect [68] moves the plasma at higher altitudes (where recombination is less), where the plasma “spreads” along the magnetic field further from the magnetic equator (under the influence of gravity and pressure). This intensifies and poleward shifts the equatorial anomaly crests. During the main phase of the geomagnetic storm in November 2025, the equatorial anomaly crest intensified up to 175 TECU and shifted poleward by 8–10°. Such crucial intensifications and shifts were recorded during the St. Patrick’s Day 2015 and the Hallowing 2003 geomagnetic storms [45,69].

During geomagnetic storms, ionospheric scintillations caused by radio signals scattering on small-scale ionospheric irregularities decrease kinematic PPP mode positioning accuracy [70]. During the main phase of the geomagnetic storm on 12 November 2025, positioning errors increased up to 2–3 m in receivers located at high latitudes and in regions affected by the super equatorial bubble. During the St. Patrick’s Day 2015 storm [70,71], positioning errors increased up to 0.7 m, while, during the May 2024 extreme geomagnetic storm, they increased up to 1 m [72]. Zakharenkova and Cherniak [73] found that, during the geomagnetic storm on 7–8 September 2017, positioning accuracy degraded at many GPS receivers in North America, particularly within the 25–35° N area affected by the movement of the equatorial bubble. In the American sector, positioning errors increased to several meters compared to calm conditions, when the error is usually up to several decimeters.

During the main phase of the 12 November 2025 geomagnetic storm, ionospheric scatter was observed at latitudes of up to 59° N and 62° N on the EKB (~55° MLAT) and MGW (~56° MLAT) radars, respectively. Based on the MGW radar data, the region of intense small-scale irregularities shifted equatorward during 06:00–18:00 UT on 12 November. Individual moments of ionospheric scatter observations correlated with the bursts in the AE index (Figure 6a) that characterized the auroral electrojet intensity. The dynamics of the auroral oval equatorial boundary on the ROTI keogram for the European–Asian sector (Figure 9, bottom panel) correlated with observations of the lowest latitude of ionospheric scatter recorded by the radars (Figure 16). During the 25–26 August 2018 severe geomagnetic storm, the dynamics of the auroral oval boundary based on ROTI also correlated with ionospheric scatter recorded by high-frequency radar in the eastern (80° W) and western (120° W) parts of North America, as well as in the Asian sector (60° E) [74].

During the main and recovery phases of the storm, the loss of the regular ground scatter was due to both a general decrease in the electron density and an increase in the maximum height of the ionosphere within the field of view of both high-frequency radars. Absorption also affected radio wave propagation. The EKB radar recorded a decrease in the noise level (relative to the median for calm conditions) during the main phase of the storm (02–08 UT on 12 November). Between 08 and 16 UT on 12 November, the noise level fluctuated, featuring multiple increases and decreases. The decrease in the noise level indicates an increase in the absorption of high-frequency radio waves, which is associated with additional ionization in the lower ionosphere [75]. Increased absorption may indicate a displacement of the boundary of the diffuse precipitation zone to latitudes where the EKB is located. Decreased electron density, increased maximum height of the ionosphere and increased absorption lead to disruptions in radio communication and radar systems [76].

An increase in the solar wind pressure and velocity, as well as in the interplanetary magnetic field (especially the negative Bz component), enhances the magnetospheric convection electric field [77]. This displaces the main ionospheric trough and the diffuse precipitation zone towards lower latitudes. Using Burk’s model [78], we estimated that the electric field increased, ranging within 2–3 mV/m during the storm main phase (00–11 UT on 12 November). The electric field also increased during the second decrease in the SYM-H index on 13 November (00–08 UT), reaching ~1 mV/m. During these periods, the main ionospheric trough and the precipitation zone (auroral oval) shifted southwards, entering the field of view of the high-frequency radars. The full picture of the ionospheric response (ROTI and adjusted TEC) and the dynamics of the 3D positioning errors during the 12 November 2025 Ugly Duckling geomagnetic storm can be found in the Supplementary Materials of this article.

## 5. Conclusions

We studied the effects of the 12 November 2025 Ugly Duckling geomagnetic storm on near-Earth space, combining data from GNSS networks, ionosondes, optical instruments, high-frequency radars (SuperDARN and SuperDARN-like), cosmic ray neutron monitors and ACE spacecraft. This storm is the third most intense storm in solar cycle 25 after the May 2024 extreme geomagnetic storm and the severe storm in October 2024. The main conclusions are as follows:

1. A series of three powerful X-class solar flares, an X1.7 (9 November 2025), an X1.2 (10 November 2025), and an X5.1 (11 November 2025) solar flare, originated from the same active region in November 2025. Each flare was accompanied by a coronal mass ejection (CME). The complex shock-ICME structure arrived at Earth, triggering a severe geomagnetic storm (Kp = 9-, Dst = −217 nT and SYM-H = −254 nT). On 13 November 2025, during the storm’s recovery phase, the CME-related shock from the most powerful X5.1 solar flare arrived, causing a renewed drop in Dst and a second enhancement of geomagnetic activity.
2. The auroral oval expanded equatorward, reaching latitudes of ~35° N in the American sector and ~45° N in the European sector. The ROTI reached 1.5–2 TECU/min.
3. We recorded a super equatorial plasma bubble almost reaching the auroral oval boundary in the American sector. The equatorial anomaly crests intensified up to 175 TECU and shifted poleward by 8–10°.
4. During the main phase, the critical frequency (foF2) exhibited a strong negative disturbance (−50%) at mid-latitudes, followed by an unusually prolonged and intense positive phase exceeding +100%.
5. The geomagnetic storm was accompanied by a Forbush effect (decrease in the intensity of cosmic rays), against which a ground-level enhancement (GLE77) was recorded.
6. Mid-latitude auroras were recorded in the OI 630 nm airglow. In addition, the SAR arc was recorded; its width changed from ~40 degrees to ~20 degrees during the geomagnetic storm.
7. At high latitudes, high-frequency radio waves suffered from absorption: the blackouts occurred in 80% or more measurements. At mid-latitudes, the blackouts occurred in fewer than 3% of measurements.
8. GPS positioning errors in PPP mode increased to 2–3 m at high latitudes and in regions affected by the super equatorial bubble. This displacement of the increased positioning errors correlated to the expansion of the boundaries of the auroral oval and the increased ionization.

## Figures and Tables

**Table 1:** Strong flares in active region 14274 in November 2025.

Day of Month	Time of Maximum, UT	X-Ray Class	Arrival Shock Waves in Lagrange Point L1 ^1^ (ACE)	Arrival Shock Waves to the Earth’s Bow Shock Nose (Estimated)	Speed of CME-Related Shock ^2^, km/s
03	10:11	M5.0			
04	17:34	X1.8			
05	11:19	M7.45			
05	22:07	M8.6			
**09**	**07:35**	**X1.7**	**11 November at 22:15 UT**	**11 November at 23:15 UT**	**804**
**10**	**09:19**	**X1.2**	**11 November at 23:38 UT**	**12 November at 00:20 UT**	**1320**
**11**	**10:04**	**X5.1**	**12 November at 18:53 UT**	**12 November at 19:45 UT**	**1350**
14	08:30	X4.0			

^1^ L1 is the first Lagrange point (or libration point) in the Sun–Earth system, which is balanced by the gravitational attraction of the two objects and lies on a straight line between them. ^2^ The speed of the CME-related shock is estimated based on the metric type II solar radio bursts [32].

## Data Availability

The data presented in this study are openly available in the SIMuRG repository (https://simurg.iszf.irk.ru, accessed on 2 February 2026); the input data in RINEX format are openly available in a number of repositories.

## Supplementary Materials

The following supporting information can be downloaded at https://www.mdpi.com/article/10.3390/s26051490/s1, Video S1: Global dynamics for ROTI, adjusted TEC and GPS PPP errors during the 12 November 2025 Ugly Duckling geomagnetic storm.

## Abbreviations

The following abbreviations are used in this manuscript:

ACE	Advanced Composition Explorer
AR	Active Regions
CME	Coronal Mass Ejection
GAMP	GNSS Analysis Software for Multi-Constellation and Multi-Frequency Precise Positioning
GIM	Global Ionospheric Maps
GIRO	Global Ionospheric Radio Observatory
GLE	Ground-Level Enhancement
GNSS	Global Navigation Satellite Systems
GOES	Geostationary Operational Environmental Satellite
CORS	Continuously Operating Reference Stations
GPS	Global Positioning System
Dst	Disturbance Storm Time Index
foF2	Critical Frequency of the F2 Layer Maxima
hmF2	Height of the F2 Layer Maxima
ICME	Interplanetary Coronal Mass Ejection
IMF	Interplanetary Magnetic Field
ISTP SB RAS	Institute of Solar-Terrestrial Physics of Siberian Branch of Russian Academy of Sciences
L1	The First Lagrange Point
MIT	Main Ionospheric Trough
MLAT	Magnetic Latitude
NmF2	Electron Density of the F2 Layer Maxima
NOAA	National Oceanic and Atmospheric Administration
pfu	Proton Flux Unit
PPP	Precise Point Positioning
ROTI	Rate Of TEC Index
SAR arc	Stable Auroral Red Arc
SECIRA	Network of Coherent Ionospheric Radars
SIDC	Solar Influences Data Analysis Center
SIMuRG	System for Ionosphere Monitoring and Research from GNSS
SDO	Solar Dynamic Observatory
SuperDARN	Super Dual Auroral Radar Network
TEC	Total Electron Content
TECU	Total Electron Content Unit

## References

[1] Danilov A., Lastovicka J. (2001). Effects of Geomagnetic Storms on the Ionosphere and Atmosphere. Int. J. Geomagn. Aeron..

[2] Mendillo M. (2006). Storms in the Ionosphere: Patterns and Processes for Total Electron Content. Rev. Geophys..

[3] Cander L.R. (2019). Ionospheric Storm Morphology. Ionospheric Space Weather; Springer Geophysics.

[4] Yeh K.C., Liu C.H. (1982). Radio Wave Scintillations in the Ionosphere. Proc. IEEE.

[5] Pilipenko V. (2021). Space Weather Impact on Ground-Based Technological Systems. Sol.-Terr. Phys..

[6] Berngardt O. (2017). Space Weather Impact on Radio Device Operation. Sol.-Terr. Phys..

[7] Demyanov V., Yasyukevich Y. (2021). Space Weather: Risk Factors for Global Navigation Satellite Systems. Sol.-Terr. Phys..

[8] Luo X., Gu S., Lou Y., Xiong C., Chen B., Jin X. (2018). Assessing the Performance of GPS Precise Point Positioning Under Different Geomagnetic Storm Conditions during Solar Cycle 24. Sensors.

[9] Béland J., Small K., Daglis I.A. (2005). Space Weather Effects on Power Transmission Systems: The Cases of Hydro-Québec and Transpower New ZealandLtd. Effects of Space Weather on Technology Infrastructure.

[10] Thaduri A., Galar D., Kumar U. (2020). Space Weather Climate Impacts on Railway Infrastructure. Int. J. Syst. Assur. Eng. Manag..

[11] Wang C., Richardson J.D., Burlaga L. (2001). Propagation of the Bastille Day 2000 CME Shock in the Outer Heliosphere. Sol. Phys..

[12] Jacobsen K.S., Andalsvik Y.L. (2016). Overview of the 2015 St. Patrick’s Day Storm and Its Consequences for RTK and PPP Positioning in Norway. J. Space Weather Space Clim..

[13] Tsurutani B.T., Judge D.L., Guarnieri F.L., Gangopadhyay P., Jones A.R., Nuttall J., Zambon G.A., Didkovsky L., Mannucci A.J., Iijima B. (2005). The October 28, 2003 Extreme EUV Solar Flare and Resultant Extreme Ionospheric Effects: Comparison to Other Halloween Events and the Bastille Day Event. Geophys. Res. Lett..

[14] Pulkkinen A., Lindahl S., Viljanen A., Pirjola R. (2005). Geomagnetic Storm of 29–31 October 2003: Geomagnetically Induced Currents and Their Relation to Problems in the Swedish High-voltage Power Transmission System. Space Weather.

[15] Shen C., Xu M., Wang Y., Chi Y., Luo B. (2018). Why the Shock-ICME Complex Structure Is Important: Learning from the Early 2017 September CMEs. Astrophys. J..

[16] Ruffa J.A., Bay M., Ward D.K., Gonzales P.J., Bartusek L.M., Pesnell W.D. (2012). NASA’s Solar Dynamics Observatory (SDO): A Systems Approach to a Complex Mission. Proceedings of the 2012 IEEE Aerospace Conference, Big Sky, MT, USA, 3–10 March 2012.

[17] Berghmans D., Hochedez J.F., Defise J.M., Lecat J.H., Nicula B., Slemzin V., Lawrence G., Katsyiannis A.C., Der Linden R.V., Zhukov A. (2006). SWAP Onboard PROBA 2, a New EUV Imager for Solar Monitoring. Adv. Space Res..

[18] King J.H., Papitashvili N.E. (2005). Solar Wind Spatial Scales in and Comparisons of Hourly Wind and ACE Plasma and Magnetic Field Data. J. Geophys. Res..

[19] Yasyukevich Y.V., Kiselev A.V., Zhivetiev I.V., Edemskiy I.K., Syrovatskii S.V., Maletckii B.M., Vesnin A.M. (2020). SIMuRG: System for Ionosphere Monitoring and Research from GNSS. GPS Solut..

[20] Pi X., Mannucci A.J., Lindqwister U.J., Ho C.M. (1997). Monitoring of Global Ionospheric Irregularities Using the Worldwide GPS Network. Geophys. Res. Lett..

[21] Li J., Ma G., Maruyama T., Wan Q., Fan J., Zhang J., Wang X. (2022). ROTI Keograms Based on CMONOC to Characterize the Ionospheric Irregularities in 2014. Earth Planets Space.

[22] Padokhin A.M., Andreeva E.S., Nazarenko M.O., Kalashnikova S.A. (2022). Phase-Difference Approach for GNSS Global Ionospheric Total Electron Content Mapping. Radiophys. Quantum Electron..

[23] Liu Q., Hernández-Pajares M., Lyu H., Goss A. (2021). Influence of Temporal Resolution on the Performance of Global Ionospheric Maps. J. Geod..

[24] Roma-Dollase D., Hernández-Pajares M., Krankowski A., Kotulak K., Ghoddousi-Fard R., Yuan Y., Li Z., Zhang H., Shi C., Wang C. (2018). Consistency of Seven Different GNSS Global Ionospheric Mapping Techniques during One Solar Cycle. J. Geod..

[25] Dow J.M., Neilan R.E., Rizos C. (2009). The International GNSS Service in a Changing Landscape of Global Navigation Satellite Systems. J. Geod..

[26] Zhou F., Dong D., Li W., Jiang X., Wickert J., Schuh H. (2018). GAMP: An Open-Source Software of Multi-GNSS Precise Point Positioning Using Undifferenced and Uncombined Observations. GPS Solut..

[27] Ratovsky K.G., Medvedev A.V., Tolstikov M.V., Kushnarev D.S. (2008). Case Studies of Height Structure of TID Propagation Characteristics Using Cross-Correlation Analysis of Incoherent Scatter Radar and DPS-4 Ionosonde Data. Adv. Space Res..

[28] Lukovnikova A. (2015). Present State of Cosmic Ray Stations of the Institute of Solar-Terrestrial Physics of Siberian Branch of the Russian Academy of Sciences (ISTP SB RAS). J. Phys. Conf. Ser..

[29] Vasilyev R., Artamonov M., Beletsky A., Zorkaltseva O., Komarova E., Medvedeva I., Mikhalev A., Podlesny S., Ratovsky K., Syrenova T. (2020). Scientific Goals of Optical Instruments of the National Heliogeophysical Complex. Sol.-Terr. Phys..

[30] Berngardt O., Kurkin V., Kushnarev D., Grkovich K., Fedorov R., Orlov A., Harchenko V. (2020). ISTP SB RAS Decameter Radars. Sol.-Terr. Phys..

[31] Clette F., Lefèvre L. (2015). SILSO Sunspot Number.

[32] Gopalswamy N., Thompson W.T., Davila J.M., Kaiser M.L., Yashiro S., Mäkelä P., Michalek G., Bougeret J.-L., Howard R.A. (2009). Relation Between Type II Bursts and CMEs Inferred from STEREO Observations. Sol. Phys..

[33] Papitashvili N.E., King J.H. (2020). OMNI 5-Min Data Set. Space Physics Data Facility. https://spase-metadata.org/NASA/NumericalData/OMNI/HighResolutionObservations/Version1/PT5M.

[34] Richardson I.G., Cane H.V. (1995). Regions of Abnormally Low Proton Temperature in the Solar Wind (1965–1991) and Their Association with Ejecta. J. Geophys. Res..

[35] Yermolaev Y.I., Nikolaeva N.S., Lodkina I.G., Yermolaev M.Y. (2009). Catalog of Large-Scale Solar Wind Phenomena during 1976–2000. Cosm. Res.

[36] Richardson I.G., Cane H.V. (2010). Near-Earth Interplanetary Coronal Mass Ejections During Solar Cycle 23 (1996–2009): Catalog and Summary of Properties. Sol. Phys..

[37] Matzka J., Stolle C., Yamazaki Y., Bronkalla O., Morschhauser A. (2021). The Geomagnetic *Kp* Index and Derived Indices of Geomagnetic Activity. Space Weather.

[38] Sugiura M. (1964). Hourly Values of Equatorial Dst for the IGY.

[39] Wanliss J.A., Showalter K.M. (2006). High-resolution Global Storm Index: *Dst* versus SYM-H. J. Geophys. Res..

[40] Rassoul H.K., Rohrbaugh R.P., Tinsley B.A., Slater D.W. (1993). Spectrometric and Photometric Observations of Low-latitude Aurorae. J. Geophys. Res..

[41] Mikhalev A. (2019). Mid-Latitude Aurora in Solar Cycles 23–24 from Observations in the South of Eastern Siberia. Sol.-Terr. Phys..

[42] Deminov M.G., Shubin V.N. (2018). Empirical Model of the Location of the Main Ionospheric Trough. Geomagn. Aeron..

[43] Gonzalez-Esparza J.A., Sanchez-Garcia E., Sergeeva M., Corona-Romero P., Gonzalez-Mendez L.X., Valdes-Galicia J.F., Aguilar-Rodriguez E., Rodriguez-Martinez M., Ramirez-Pacheco C., Castellanos C.I. (2024). The Mother’s Day Geomagnetic Storm on 10 May 2024: Aurora Observations and Low Latitude Space Weather Effects in Mexico. Space Weather.

[44] Li Y., Zhang H., Xu F., Ding Q., Tang L. (2025). Super Equatorial Plasma Bubbles Observed Over South America During the October 10 and 11, 2024 Strong Geomagnetic Storm. IEEE Geosci. Remote Sens. Lett..

[45] Astafyeva E., Zakharenkova I., Förster M. (2015). Ionospheric Response to the 2015 St. Patrick’s Day Storm: A Global Multi-instrumental Overview. JGR Space Phys..

[46] Yasyukevich Y.V., Vasiliev R.V., Rubtsov A.V., Alsatkin S.S., Artamonov M.F., Beletsky A.B. (2025). Extreme Magnetic Storm of May 10–19, 2024: Coupling Between Neutral and Charged Components of the Upper Atmosphere and the Effect on Radio Systems. Dokl. Earth Sci..

[47] Cherniak I., Zakharenkova I. (2015). Dependence of the High-Latitude Plasma Irregularities on the Auroral Activity Indices: A Case Study of 17 March 2015 Geomagnetic Storm. Earth Planet Space.

[48] Yao Y., Liu L., Kong J., Zhai C. (2016). Analysis of the Global Ionospheric Disturbances of the March 2015 Great Storm. JGR Space Phys..

[49] Ledvina B.M., Makela J.J., Kintner P.M. (2002). First Observations of Intense GPS L1 Amplitude Scintillations at Midlatitude. Geophys. Res. Lett..

[50] Fremouw E.J., Secan J.A., Lansinger J.M. (1985). Spectral Behavior of Phase Scintillation in the Nighttime Auroral Region. Radio Sci..

[51] Chernyshov A.A., Klimenko M.V., Nosikov I.A., Borchevkina O.P., Timchenko A.V., Efishov I.I., Sinevich A.A., Ryakhovsky I.A., Yakimova G.A., Bessarab F.S. (2025). Effects in the Upper Atmosphere and Ionosphere in the Subauroral Region during Victory Day 2024 Geomagnetic Storm (May 10–12, 2024). Adv. Space Res..

[52] Danilchuk E., Yasyukevich Y., Vesnin A., Klyusilov A., Zhang B. (2025). Impact of the May 2024 Extreme Geomagnetic Storm on the Ionosphere and GNSS Positioning. Remote Sens..

[53] Luo X., Aa E., Wang X., Luo B. (2026). Strong Longitudinal and Latitudinal Differences of Ionospheric Responses in North American and European Sectors During the 10–11 October 2024 Geomagnetic Storm. Remote Sens..

[54] Sahai Y., Fagundes P.R., Bittencourt J.A. (2000). Transequatorial F-Region Ionospheric Plasma Bubbles: Solar Cycle Effects. J. Atmos. Sol.-Terr. Phys..

[55] Tulasi Ram S., Ajith K.K., Yamamoto M., Otsuka Y., Yokoyama T., Niranjan K., Gurubaran S. (2015). Fresh and Evolutionary-type Field-aligned Irregularities Generated near Sunrise Terminator Due to Overshielding Electric Fields. JGR Space Phys..

[56] Huang F., Lei J., Xiong C., Zhong J., Li G. (2021). Observations of Equatorial Plasma Bubbles during the Geomagnetic Storm of October 2016. Earth Planet. Phys..

[57] Ma G., Maruyama T. (2006). A Super Bubble Detected by Dense GPS Network at East Asian Longitudes. Geophys. Res. Lett..

[58] Zakharenkova I., Cherniak I., Krankowski A., Valladares C.E., De La Jara Sanchez C. (2025). On Detection of Super Equatorial Plasma Bubbles in the American Sector During the 10–11 October 2024 Geomagnetic Storm. JGR Space Phys..

[59] Pulinets S., Kotonaeva N., Depuev V., Tsybulya K. (2026). Ionospheric Response to the Geomagnetic Storm of 12–14 November 2025, Based on Multi-Parameter Analysis of Data from the LAERT Topside Sounder. Atmosphere.

[60] Pulinets S., Tsybulya K., Depuev V., Danilov I., Pulinets M. (2026). Ionospheric Topside Sounding Revival. Adv. Space Res..

[61] Suraina, Rakhman A., Abadi P., Kilowasid L.O.M.M., Putra A.Y., Perwitasari S., Irnaka T.M. (2025). Pre-Sunrise Equatorial Plasma Bubble Over Indonesia During the 11 May 2024 Super Geomagnetic Storm. Earth Space Sci..

[62] Cherniak I., Zakharenkova I. (2022). Development of the Storm-Induced Ionospheric Irregularities at Equatorial and Middle Latitudes During the 25–26 August 2018 Geomagnetic Storm. Space Weather.

[63] Luan X., Huang C., Lu G., Zhang Y., Paxton L.J. (2021). Equatorial Ionization Anomaly Variations During Geomagnetic Storms. Geophysical Monograph Series.

[64] Kelley M.C. (2009). The Earth’s Ionosphere: Plasma Physics and Electrodynamics.

[65] Tsurutani B.T., Verkhoglyadova O.P., Mannucci A.J., Saito A., Araki T., Yumoto K., Tsuda T., Abdu M.A., Sobral J.H.A., Gonzalez W.D. (2008). Prompt Penetration Electric Fields (PPEFs) and Their Ionospheric Effects during the Great Magnetic Storm of 30–31 October 2003. J. Geophys. Res..

[66] Blanc M., Richmond A.D. (1980). The Ionospheric Disturbance Dynamo. J. Geophys. Res..

[67] Bhattacharyya A. (2022). Equatorial Plasma Bubbles: A Review. Atmosphere.

[68] Tsurutani B., Mannucci A., Iijima B., Abdu M.A., Sobral J.H.A., Gonzalez W., Guarnieri F., Tsuda T., Saito A., Yumoto K. (2004). Global Dayside Ionospheric Uplift and Enhancement Associated with Interplanetary Electric Fields. J. Geophys. Res..

[69] Mannucci A.J., Tsurutani B.T., Iijima B.A., Komjathy A., Saito A., Gonzalez W.D., Guarnieri F.L., Kozyra J.U., Skoug R. (2005). Dayside Global Ionospheric Response to the Major Interplanetary Events of October 29–30, 2003 “Halloween Storms”. Geophys. Res. Lett..

[70] Yang Z., Morton Y.T.J., Zakharenkova I., Cherniak I., Song S., Li W. (2020). Global View of Ionospheric Disturbance Impacts on Kinematic GPS Positioning Solutions During the 2015 St. Patrick’s Day Storm. JGR Space Phys..

[71] Nie W., Rovira-Garcia A., Li M., Fang Z., Wang Y., Zheng D., Xu T. (2022). The Mechanism for GNSS-Based Kinematic Positioning Degradation at High-Latitudes Under the March 2015 Great Storm. Space Weather.

[72] Yang Z., Morton Y.T.J. (2025). Impacts of the May 2024 Extreme Geomagnetic Storm on Global High-Accuracy GPS Positioning Solutions. Space Weather.

[73] Zakharenkova I., Cherniak I. (2021). Effects of Storm-Induced Equatorial Plasma Bubbles on GPS-Based Kinematic Positioning at Equatorial and Middle Latitudes during the September 7–8, 2017, Geomagnetic Storm. GPS Solut..

[74] Astafyeva E., Yasyukevich Y.V., Maletckii B., Oinats A., Vesnin A., Yasyukevich A.S., Syrovatskii S., Guendouz N. (2022). Ionospheric Disturbances and Irregularities During the 25–26 August 2018 Geomagnetic Storm. JGR Space Phys..

[75] Berngardt O.I., Ruohoniemi J.M., Nishitani N., Shepherd S.G., Bristow W.A., Miller E.S. (2018). Attenuation of Decameter Wavelength Sky Noise during X-Ray Solar Flares in 2013–2017 Based on the Observations of Midlatitude HF Radars. J. Atmos. Sol.-Terr. Phys..

[76] Ponomarchuk S., Zolotukhina N., Kurkin V., Belinskaya A., Grozov V., Oinats A., Poddelsky A., Podlesnyi A., Cedrik M. (2025). Effects of the May 10–13, 2024 Magnetic Storm in the Asian Region of Russia from Ionospheric Sounding with a Continuous Chirp Signal. Sol.-Terr. Phys..

[77] Nishida A. (1978). Geomagnetic Diagnosis of the Magnetosphere.

[78] Burke W.J., Huang C.Y., Marcos F.A., Wise J.O. (2007). Interplanetary Control of Thermospheric Densities during Large Magnetic Storms. J. Atmos. Sol.-Terr. Phys..

